# Early transfer of cases from emergency(ER) to ICU (≤1 hour) - does it really make a big difference in outcome? an analysis

**DOI:** 10.1186/2197-425X-3-S1-A363

**Published:** 2015-10-01

**Authors:** A Kar, A Datta, A Ahmed

**Affiliations:** Medica Institute of Critical Care (MICC), Medica Superspecialty Hospital, Kolkata, India

## Introduction

Turnaround Time (TAT) of patients shifted to ICU from Emergency department is of outmost importance as delay in transfer is associated with increasing mortality as shown in different studies. The longer the patient stays in ER the more likely their care could be compromised.

## Objectives

Our aim of the study was to determine whether early transfer (≤1 hour) from Emergency department (ER) to ICU do make a significant difference in outcome. This was done in a Level 3 Care ICU of a tertiary care hospital in Kolkata.

## Methodology

All cases transferred in a level 3 Care ICU from ER over a period of one year were included and TAT documented. Patients were divided into Control group (Group A with TAT≤1 hour) and other groups (Group B with TAT>1 hour≤3 hours, Group C with TAT >3 hours≤6 hours, Group D with TAT>6 hours). Outcome assessment was done using APACHE IV model on admission to ICU. Predicted Mortality Rate (PMR), Standardized Mortality Ratio (SMR) was calculated and observed death in each group was documented. Comparison between Control group and other groups was done using unpaired Student t test and p value < 0.05 was considered significant to see whether there is significant difference in outcome based on mortality (SMR).

## Results

Group A (TAT≤1 hour) had 463 patients, Predicted Mortality Mean (15.35) [Median 4.47], Observed deaths 67, SMR was 0.94 (CI .74-1.19). Group B (TAT> 1hour≤3 hours) had 688 patients, Predicted Mortality Mean (12.28) [Median 4.68], Observed deaths 81, SMR was 0.96 (CI .77-1.19),]. Group C (TAT>3 hours≤6 hours) had 301 patients, Predicted Mortality Mean (8.38) [Median 2.24], Observed deaths 23, SMR was 0.91 (CI .59-1.34). Group D (TAT>6 hours) had 284 patients, Predicted Mortality Mean (5.98) [Median 1.69], Observed deaths 16, SMR was 0.94 (CI .56-1.50). **While analyzing between Control group (Group A) with other groups (Group B, Group C, Group D) PMR was significantly higher in Group A versus Group B (p = 0.014), versus Group C (p < .0001) and versus Group D (p < .0001). SMR was not significantly different between Control and other groups.**

## Conclusions

In our set up SMR was not significantly different between Control and other groups which were attributed to availability of adequate resources/ Equipment, early involvement of multidisciplinary team and on-going quality of care. Early transfer from ER to ICU is an important quality parameter but other aspects of care should also be looked upon while relating to outcome (mortality).
